# Influence of Marriages of Consanguinity upon Offspring

**Published:** 1857-09

**Authors:** 


*Influence of Marriages of Consanguinity upon Offspring.*—We would beg the attention of our readers to the subjoined Circular. Dr. Bemiss has already given to the above subject considerable attention, and, in consequence of his publications relating thereto, he was appointed, at the last meeting of the Am. Med. Association, to collect facts and report at the meeting in May next.

Circulars of this kind, as a rule, have hitherto met with very little response from the profession. It is evident that statistical data for an investigation of a problem like that assigned to Dr. B., can only be acquired by means of the coöperation of a number of persons whose interest in science is sufficient to lead them to take some pains in furtherance of its objects. With the data which may be contributed, Dr. B. will be able to arrive at important results and present to the association a valuable report. Let all our readers send an account of all the instances of marriages among blood-relations within their knowledge, answering carefully the questions given in the circular. Even a single instance transmitted by every medical reader of this Journal, would, in the aggregate, form a number, the analysis of which would lead to valuable results.

We would call attention particularly to the request of Dr. Bemiss that instances of such marriages in which no defective issue results, or which are sterile, may be sent as well as instances of an opposite description. To determine fairly the influence of consanguinity, it is plain that instances of the former as well as the latter must be obtained.

We trust most sincerely that the present effort to obtain statistical data will prove an exception to the rule as regards the distribution of circulars hitherto; and that Dr. Bemiss will find himself in possession of abundant data to arrive at important conclusions. *

LOUISVILLE, Ky., July 16, 1857.

DEAR SIR:

May I solicit your coöperation in collecting material for a report I am to present to the next American Medical Association on the "Influence of Marriages of Consanguinity upon offspring." I wish to make my report exclusively statistical, and if each of the medical gentlemen to whom I address calls for assistance, will contribute even a small amount of aid, I will be enabled to sum up results enough to justify very positive conclusions upon this subject.

If instances of such marriages have occurred under your notice, will you oblige me my sending the results as carefully analyzed as your knowledge of circumstances will permit?

The points of which I wish more especially to be informed, are:

1. The degree of consanguinity of parties, (whether first, second, third or fourth cousins; or as in rore instances, uncle and niece.)

2. The date (approximative) of marriage.

3. The number, sex and *condition* of children born to each marriage.

4. The number of children dead, cause of death, and age when known.

5. The constitution, temperament and occupation of parents, with any habits or circumstances calculated either to favor or retard the normal development of offspring.

I wish my report to be entirely unprejudiced; my only aim is *truth*, and I desire those instances of such marriages where no defective issue results, or which are sterile, as carefully sought out and reported as the opposite; and whenever the defects of offspring may be reasonably referred to other causes than consanguinity, I wish the facts distinctly set forth.

I am well aware that investigations is in these cases often foiled by the sensitiveness of parents upon the subject of kinship, but in such instances information as circumstantial and reliable may often be obtained from friends of the family.

I trust, My Dear Sir, I will not be accused of making undue demands upon your time and patience in these requests, such is my anxiety to furnish a comprehensive report, and one that will embody a great amount of new and valuable information, that I will await your answer with much solicitude, and cherish grateful recollections of all who consent to become my co-laborers.

If your engagements should prevent your own participation in these enquiries, would you be so kind as to entrust them to an intelligent student, or any reliable person whom you may select?

I wish to get in my statistics by the first of the ensuing year, so as to tabulate them, but would be gratified to hear from you at an earlier period.

Very respectfully,

S. M. Bemiss.

P. S. Names of parties not desired, as all personal allusion will be avoided in my report.

---

*Science and Good Sense.*—The following extract is from a lecture introductory to a clinical course at the Hospital La Riboisière, at Paris, by Dr.

Pidoux.* "Science is less difficult than good sense. Science takes the physical signs of disease for the disease itself. She confounds the morbid anatomy of the body with pathology; and, having thus destroyed disease, she reänimates it by a *Deus ex machina*, by investing it with a soul or a vital principle which seem to have been invented expressly for the protection of anatomical medicine. Montpellier lends for a moment her life without organs to the organs without life of Paris, and from these two abstractions is formed a scholastic entity very convenient for argument." *

